# The J Technique for Barbed Suture Closure

**DOI:** 10.1016/j.atssr.2022.11.009

**Published:** 2022-11-24

**Authors:** Lowell Leow, Emily Chok, Alexandra Chua, Yue Li, Oon Cheong Ooi, Harish Mithiran, Jianye Chen, John K.C. Tam

**Affiliations:** 1 Department of Cardiac, Thoracic and Vascular Surgery, National University Heart Centre Singapore, Singapore; 2 Ethicon Singapore, Johnson & Johnson; 3 Neumark Lung & Chest Surgery Centre, Gleneagles Hospital, Singapore

## Abstract

We share our novel technique using barbed sutures for subcuticular purse-string suture closure during chest tube insertion and removal.

We present our J technique using barbed sutures to secure and to act as the purse string for chest drains (Supplemental Video). This technique is best employed for uniportal video-assisted thoracoscopic surgical cases in which the chest drains traverse the uniportal incision. It can be used effectively for up to 2 drains. We demonstrate the use of this technique for barbed sutures that are single ended, that is, having only 1 needle, as well as for double-ended sutures, which are armed with a surgical needle on each end.

## Technique

### Sutures

We use undyed Monocryl or PGA-PCL sutures for our patients in the subcuticular layer to achieve the best cosmetic result. These sutures are absorbable, have a strength retention profile of 14 days, and are fully absorbed after approximately 90 days. The barbed sutures used in our usual practice are Stratafix Monocryl Plus 3-0 and Stratafix PGA-PCL 3-0. These sutures have significantly more points of fixation than traditional sutures, making it easier to manage tension on every pass without the need for tying knots. However, for easy visibility for this video demonstration, we use dyed Stratafix PDS Plus sutures instead.

### Insertion Technique

After routine surgery, the chest drain is inserted through the wound directed toward the apex or surgical bed per the surgeon’s preference. The lungs are reinflated under direct vision, and the chest drain is anchored with silk in regular fashion. The internal fascial layer is closed in routine fashion. After this, a single- or double-ended suture is chosen for skin and drain purse string. We demonstrate the application of the J technique for 1 and 2 chest drains.

### Single-Ended Suture

To begin with a single-ended suture, a horizontal stitch is placed and anchored through the loop at the end of the barbed suture. The barbed suture is then passed circumferentially around the chest drain to create a purse string for skin apposition during removal. The stitch ends on the same side as the first stitch and out through the skin. This end is pulled during removal of the chest drain to close the purse string. The rest of the wound is closed in routine fashion with subcuticular stitches using the remnant barbed sutures.

### Double-Ended Suture

For double-ended sutures, no anchoring knot is necessary, and the stitch can be started toward the middle of the wound. A subcuticular stitch is run toward the tube, and a purse string is created in similar fashion around the chest drain. The opposite needle is used to complete the wound closure in subcuticular fashion.

### Two Chest Drains

For 2 chest drains, the barbed suture is started in the center of the wound. Subcuticular stitches are made simultaneously toward both chest drains. Once at the chest drain, the barbed suture is passed circumferentially to create the purse string. The stitch ends on the same side as the initial purse string to form an end that is left for use during removal of chest drains.

#### Troubleshooting

1. It is often easier to perform the purse-string sutures while the chest drain is free to be manipulated. We advocate withholding connection of the chest drain to the drainage device until the purse string is in.
2. If unable to get a safe purse-string bite without compromising on the chest drain, a stitch exiting in the skin can be accepted, provided the next stitch reenters at the same site. This produces a slightly worse but acceptable cosmetic result.
3. Ensure that the barbed suture does not catch the drain or anchoring stitch to avoid difficulty with removal.
4. We do not recommend the use of this technique for patients who require prolonged chest drains in situ as the dissolvable suture will lose its tensile strength over time and may snap during removal if left for more than a week.

### Removal Technique

When clinical indications for chest drain removal are met, the drain can be removed under aseptic technique. First, the anchoring stitch is cut and the drain freed. With use of a wet gauze to overlie the drain site and exerting downward pressure on the wound, the drain is removed in a swift motion under full inspiration. The barbed suture purse string is then tightened to close the skin. This produces a sealed drain site with good skin apposition. The barbed suture is then cut flush to the skin. This negates the need for hand tying of purse-string sutures and subsequent removal of stitches. This also allows the chest drain to be removed by a single operator.

#### Troubleshooting

1. In the event that the barbed suture snaps, the wet gauze exerting downward pressure helps prevent air entry into the pleura. If assistance is available, place a simple interrupted stitch using monofilament sutures to close the drain site. If assistance is not available, a temporary airtight dressing such as Tegaderm can be placed over the wound while the necessary equipment is obtained to stitch the drain site.
2. If the barbed suture is unable to be tightened, it may have been caught on another suture or structure. The barbed suture should then be cut flush to the skin and the drain site closed with an interrupted stitch in usual fashion.
3. If the chest drain is unable to be removed, the purse string may have caught the drain. In such instances, the purse string would need to be released and removed to allow safe removal of the chest drain.

## Comment

In our experience, we have been using the J technique since 2018 to good effect. The lack of need for an additional removal of stitches and equivalent cosmetic result are advantageous for our patients.

